# Comparison of the Anterior Septal Line and Mitral Isthmus Line for Perimitral Atrial Flutter Ablation Using Robotic Magnetic Navigation

**DOI:** 10.1155/2022/1793590

**Published:** 2022-02-01

**Authors:** Shipeng Dang, Ru-Xing Wang, Christian Jons, Peter Karl Jacobsen, Steen Pehrson, Xu Chen

**Affiliations:** ^1^Department of Cardiology, The Affiliated Wuxi People's Hospital of Nanjing Medical University, Qingyang Road 299, Wuxi, China; ^2^Department of Cardiology, The Heart Centre, Rigshospitalet, Copenhagen University Hospital, Inge LehmannsVej 7, Copenhagen, Denmark

## Abstract

**Background:**

Perimitral atrial flutter (PMAFL) is one of the most common macro-reentrant left atrial tachycardias. Mitral isthmus (MI) linear ablation is a common strategy for the treatment of PMAFLs, and anterior septum (AS) linear ablation has emerged as a novel ablation approach. We aimed at assessing the effectiveness of AS linear ablation using robotic magnetic navigation for PMAFL ablation.

**Methods:**

In this retrospective study, a total of 36 consecutive patients presented with AFL as the unique arrhythmia or accompanied with atrial fibrillation (AF) who underwent catheter ablation were enrolled. Patients were classified into two groups according to the different ablation strategies, the MI line group (10 patients) and the AS line group (26 patients).

**Results:**

The clinical baseline characteristics of patients in the two groups were nearly identical. There were no significant differences in procedure time (148.7 ± 46.1 vs. 123.2 ± 30.1 min, *P*=0.058) or radiofrequency ablation time (25.9 ± 11.4 vs. 23.5 ± 12.6 min) between the two groups. Fluoroscopy time was longer in the MI line group (8.0 ± 4.4 vs. 5.1 ± 2.7 min, *P*=0.024), and the acute success rate was higher in the AS line group versus the MI line group (96.2% vs. 70%, *P*=0.025). The long-term freedom from arrhythmia survival rate was higher in the AS line group (73%) than in the MI line group (40%) after a mean follow-up time of 37.4 months with a 3-month blanking period (*P*=0.049).

**Conclusions:**

AS linear ablation is an effective and safe strategy for PMAFL ablation using robotic magnetic navigation.

## 1. Introduction

Left atrial tachycardias (ATs) are primarily seen in patients after radiofrequency catheter ablation (RFCA) or surgical therapy in the left atrium (LA) and in patients with atrial myopathies [1]. The substrate for these ATs is often a scar with resultant conduction slowing [2]. These slow conduction zones form a critical isthmus in the reentrant circuit, giving rise to multiple variants of AT. The most common macro-reentrant AT in the LA is perimitral and roof-dependent reentries. These left-sided ATs are difficult to treat with antiarrhythmic drugs and often require catheter ablation to achieve rhythm control [3].

Linear ablation of the mitral isthmus (MI), connecting the lateral mitral annulus to the left inferior pulmonary vein (PV), is an established strategy in the treatment of perimitral atrial flutter (PMAFL) [4]. However, bidirectional conduction block is difficult to achieve by endocardial ablation only, possibly due to the thickness of the myocardium. Epicardial ablation, especially ablation within the CS, may be required in order to obtain a conduction block over the MI [5, 6]. As an alternative strategy to MI ablation, anterior septum (AS) linear ablation, substrate ablation, and the left atrial appendage isthmus have been introduced for PMAFL [7–9]. Among these ablation approaches, the AS line connecting the right superior pulmonary veins and mitral annulus has been explored by manual ablation, but the advantages of this new approach remain controversial [7].

Robotic magnetic navigation (RMN) technology for catheter ablation allows remote control of a magnetic catheter into target cardiac locations for precise mapping and ablation. RMN technology is proven effective and safe for ablation of a variety of arrhythmias, including atrial fibrillation (AF), typical AFL, supraventricular tachycardia, and ventricular tachycardias [10–12]. However, the performance of RMN for PMAFL ablation has not previously been investigated.

In the current study, we compared the acute procedural efficacy, safety, and long-term outcome of AS linear ablation to MI linear ablation using RMN for the treatment of PMAFL.

## 2. Methods

### 2.1. Study Population

In this retrospective study, twelve PMAFL patients and twenty-four AF patients accompanied with PMAFL underwent RFCA for PMAFL and AF using RMN from January 2013 to December 2017. AFL in each patient was documented by using 12-lead electrocardiograms (ECGs) or a Holter monitor. All patients had effective anticoagulation therapy with warfarin (target international normalized ratio (INR) of 2-3) or novel anticoagulants for more than one month. In all patients, transesophageal echocardiography was performed to exclude atrial thrombus prior to ablation. Patients were excluded from the study if they (1) were younger than 18 years of age or (2) had atrial thrombus that was detected by transesophageal echocardiography. In Denmark, a retrospective study comparing the outcome of the two groups of patients after RFCA does not require approval from the local ethical committee.

### 2.2. Procedural Preparation

After femoral vein puncture, a 6F steerable catheter (Inquiry, St Jude Medical, Inc.) and a 5F quadripolar catheter (Medtronic, Inc.) were placed in the coronary sinus (CS) and the apex of the right ventricle, respectively. Surface ECG and endocardial electrograms were continuously monitored and recorded with an EP tracer (Schwarzer Cardiotek, Inc.). A single transseptal puncture was performed under hemodynamic pressure and fluoroscopic monitoring. A single bolus of 75 IU/kg body weight of heparin was given after transseptal puncture. Additional heparin of 1,000–3000 IU was administrated every hour to achieve a target ACT of 300.

### 2.3. Index or Previous AF Ablation Strategies

All patients had previously failed treatment with antiarrhythmic drug therapy. For the AFL patients accompanied with AF, pulmonary vein antrum isolation (PVI) was performed as the initial step. Electrical isolation of the PVs was verified by repeated mapping for residual potentials around the entire circumference of the PV ostia after obtaining the sinus rhythm by ablation or electrical cardioversion. Second, atypical paroxysmal AFL was induced with programmed atrial stimuli for activation mapping. Once PMAFL was confirmed by activation mapping, the patient was included in the study.

### 2.4. Mapping and Ablation of PMAFLs

All the PMAFLs, both idiopathic and those associated with AF, were confirmed by entrainment and three-dimensional activation mapping. The entrainment criterion was that the postpacing interval subtracting the tachycardia cycle length was less than 20 ms around the mitral annulus. An LA anatomic map was acquired with the open-irrigated magnetic catheter (NaviStar/Celsius® ThermoCool®RMT, Biosense Webster, Inc.) navigated by using the Niobe system (Stereotaxis, Inc., St. Louis, MO). The activation mapping was performed during AFLs, and the reentry circuit was identified around the mitral annulus by propagation mapping.

**Figure 1:**
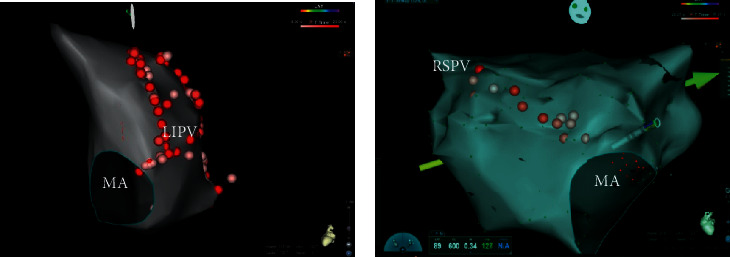
Representative picture of the MI line and anterior septal line. (a) PVI and MI linear ablation. (b) Anterior septal linear ablation.

MI linear ablation was performed in 10 patients, and AS linear ablation was performed in 26 patients during AFL or CS pacing (Figures 1(a) and 1(b)). If ablation was carried out during AFL, the first endpoint was to terminate AFL during radiofrequency energy delivery. If AFL was terminated, programmed electrical stimulation and burst atrial pacing were performed immediately after to determine whether AFL was still inducible. If AFL was terminated and was not reinducible, pacing maneuvers were performed to determine whether there was bidirectional conduction block across the ablation line. Bidirectional conduction block was also confirmed by double potentials with an interval of 100 ms or longer along the ablation line during distal CS pacing. For the MI line group, the interval of distal CS pacing to the LA appendage electrogram was longer than the interval of proximal CS pacing to the LA appendage electrogram. For the AS line group, the interval of proximal CS pacing to the LA appendage electrogram was longer than the interval of distal CS pacing to the LA appendage electrogram [7, 8]. For the MI linear ablation group, epicardial (intra-CS) ablation was not performed. If bidirectional conduction block was failed to achieve in this patient population, AS linear ablation was performed. Ablations were performed with a target temperature of not more than 43°C. Power was set at 35–40 W with a flush rate of 10 ml/min for the anterior wall and 30–35 W for the posterior wall. Fentanyl was administered in every patient for pain control.

### 2.5. Follow-Up

Patients continued antiarrhythmic and anticoagulation treatment during the blanking period of three months after the procedure. All patients were routinely evaluated in the outpatient clinic by their local cardiologist at intervals of three months and, thereafter, according to their symptoms. Twelve-lead ECGs, event recording, or Holter recordings were performed in patients with symptomatic palpitations. In addition to the scheduled follow-up visits, patients were instructed to contact a physician when suspecting arrhythmia recurrence for ECG and/or Holter documentation. Recurrence was defined as a symptomatic and/or asymptomatic AF, AT, or AFL episode > 30 seconds confirmed by ECG or Holter recordings. Long-term success was defined as no AF, AT, or AFL recurrence.

### 2.6. Statistical Analysis

Continuous variables were expressed as mean values ± standard deviation. Categorical variables were expressed as ratios and percentages. SPSS version 19.0 (SPSS Inc, Chicago, Illinois) was used for statistical analysis. Levene's test was used to check the homogeneity of variance. Normally distributed data were compared using independent Student's *t*-test. Nonnormally distributed data between the two groups were compared using the Mann–Whitney *U* test. The chi-square test was used for categorical variables. Probability of tachycardia free survival between the two groups was determined by the Kaplan–Meier estimator, and differences between the groups were tested by the Mantel–Cox (log-rank) test. All tests were performed with a two-tailed significance level of 0.05.

## 3. Results

### 3.1. Baseline Characteristics of Patients

In this retrospective study, the clinical baseline characteristics of patients in the two groups were nearly identical (summarized in Table 1). There were no statistically significant differences between the MI group and AS line group in terms of sex, age, left ventricular ejection fraction, smoking, alcohol, pacemaker, and concomitant diseases. In the MI line group, 6 patients presented with spontaneous AFL, while the other 4 patients presented with AFL and AF. They had previously undergone 1 ± 1.3 previous PVI ablations. Among the AS line group, 6 patients presented with spontaneous PMAFL and the remaining 20 patients also presented with AF. This group had previously undergone an average of 0.9 ± 0.7 previous PVI ablations.

### 3.2. Procedural Characteristics

There were no significant differences in procedure time (148.7 ± 46.1 vs. 123.2 ± 30.1 min, *P*=0.058), RFCA time (25.9 ± 11.4 vs. 23.5 ± 12.6 min, *P*=0.609), X-ray dose (9.9 ± 7.9 vs. 4.8 ± 3.1 min, *P*=0.077), heparin dose (7050.0 ± 1571.4 vs. 6807.7 ± 1428.8 U, *P*=0.660), and fentanyl dose (317.2 ± 153.1 vs. 244.3 ± 100.9 U, *P*=0.128) in these two groups. Fluoroscopy time was longer in the MI line group than the AS line group (8.0 ± 4.4 vs. 5.1 ± 2.7 min, *P*=0.024). One minor complication, a femoral vein hematoma, occurred in each group (10% vs. 3.8%, *P*=0.470) with no statistical difference (Table 2).

### 3.3. Acute and Long-Term Follow-Up Outcomes

**Figure 2:**
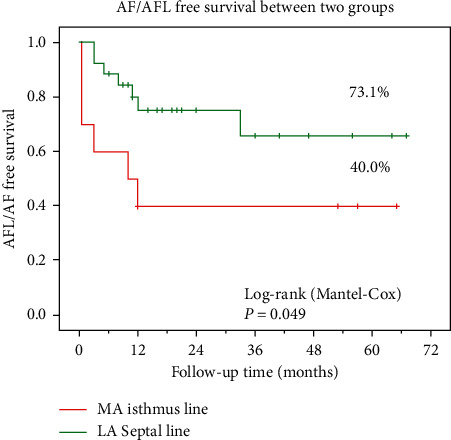
Kaplan–Meier analysis of time to AFL/AFL recurrence after the ablation procedure.

The acute success rate was lower in the MI line group than the AS line group (70% vs. 96.2%, *P*=0.025) (Table 3). For the MI line group, three patients failed to achieve bidirectional conduction block with MI linear ablation, and additional AS linear ablation was performed to achieve bidirectional conduction block. For the AS line group, one patient presented with multiple atypical AFLs, and linear ablation achieved bidirectional conduction block but without termination of tachycardia. After a mean follow-up time of 37.4 ± 20.5 months with a three-month blanking period, the recurrence rates of PMAFLs were 30.0% for the MI group and 26.9% for the AS line group, and no statistical difference was observed. Interestingly, the long-term success rate of ablation of spontaneous PMAFLs as the only arrhythmia for the MI line group (50%, 3/6) was significantly lower than that in the AS line group (100%, 6/6, *P*=0.046). Furthermore, the overall long-term arrhythmia-free survival was higher in the AS line group than the MI group (*P*=0.049) as determined by the Kaplan–Meier analysis with Mantel–Cox (log-rank) test (Figure 2).

## 4. Discussion

### 4.1. Main Findings

MI linear ablation is an established strategy in the treatment of PMAFL and a supplement to pulmonary vein isolation. In this retrospective study, we investigated the efficacy and safety of AS line and MI linear ablation for the treatment of PMAFL using RMN. The major findings are as follows: (1) AS linear ablation was a more effective approach for treating PMAFL than MI linear ablation; (2) using RMN, it was feasible to achieve bidirectional conduction block on the anterior septum. There were no serious complications in the two patient groups. This is the first study to our knowledge to evaluate the utility of RMN guidance for catheter ablation of PMAFLs.

### 4.2. Traditional Ablation of PMAFLs

Left atrial ATs are most commonly encountered not only in patients with prior LA ablation or surgery but also in patients with spontaneous fibrosis [2]. It has been reported to occur in 37% of patients as a result of AF ablations and result from focal or reentrant mechanisms in diverse locations [13]. Among different macro-reentrant LA ATs, one of the most common mechanisms is reentry along the mitral annulus, PMAFL. The management of these PMAFL patients is often a clinical challenge because they are often more symptomatic and have failed to respond to antiarrhythmic drugs [5].

Previous studies have shown that an ablation line between the left inferior pulmonary vein and mitral annulus could prevent or treat PMAFL [3, 4]. Although MI linear ablation is commonly performed in PMAFL patients, the recurrence rate of PMAFL is still high during the follow-up period [3]. An incomplete MI ablation line with subsequent gaps in the line may be proarrhythmic [3]. Despite the fact that the MI ablation line is relatively short (averaging 35 mm with a range of 15 and 52 mm), bidirectional block is difficult to achieve by endocardial ablation only. Due to the existence of epicardial connections and myocardial sleeves around the CS and the vein of Marshall [4, 6, 14, 15], epicardial ablation or ablation within the coronary sinus is often needed to achieve a bidirectional block. Ablation within the CS increases the risk of CS and circumflex artery injury, cardiac tamponade by steam pop, and thrombosis [6]. Furthermore, PMAFL with MI pseudoblock may be present after MI ablation, and a more detailed mapping in the boundaries of the ablation line or reinduction of arrhythmias may be needed to exclude residual conduction [16].

In the current study, MI linear ablation was performed in ten patients with PMAFL or with AF using RMN. Bidirectional conduction block was achieved in 7/10 (70%) patients, but was not obtained in the other three patients as epicardial and CS ablation was not performed, to avoid complications. During the follow-up period, PMAFL recurrence was found in three patients. These data suggest that RMN could be applied for MI ablation.

### 4.3. Advantages and Disadvantages of Anterior Septum (AS) Linear Ablation

Despite its proven effectiveness, the MI line is not always the optimal site for PMAFL ablation. It has been previously reported that the distribution of low-voltage zones and slow-conduction areas was mainly located on the anterior septal wall for PMAFL patients [8, 17]. Therefore, AS linear ablation emerged as an ablation approach [18]. Tzeis et al. reported that bidirectional block across the AS line was achieved in 86.1% of patients with an arrhythmia-free rate of 51.2% after 6 months of follow-up [18]. Additionally, Ammar et al. reported that acute success was achieved in 88% of patients with AS line ablation [19]. These studies all utilized manual ablation catheters. In this study, we investigated the efficacy of AS linear ablation for PMAFL with the guidance of RMN. First, our data showed that termination of PMAFL and bidirectional conduction block was achieved in 25/26 (96.2%) patients with AS linear ablation, which was significantly higher compared to the MI line group (70%). Three patients in the MI line group for which bidirectional block was not achieved did achieve a bidirectional block with subsequent AS linear ablation. These data suggest that it could be easier to achieve bidirectional conduction block in the AS line area than in the MI area. Second, long-term arrhythmia-free survival was higher in the AS line group than in the MI line group. As LA macro-reentrant ATs are most commonly perimitral and roof-dependent reentry, both MI line and roof linear ablation are needed, respectively. However, AS linear ablation could treat perimitral and roof-dependent right PV reentry AFL with one ablation lesion [2]. Third, although no serious complication occurred in either group, we propose that AS linear ablation should be safer than MI linear ablation as no ablation in the CS or epicardial ablation was needed. In summary, AS linear ablation may be a good strategy for perimitral reentry and roof-dependent reentry LA macro-reentrant ATs.

### 4.4. RMN for PMAFL Ablation

Atrial flutter can be divided into typical and atypical atrial flutter [20]. Typical atrial flutter is due to a macro-reentrant circuit around the cavotricuspid annulus [21]. The cavotricuspid isthmus, often constituting slow conduction, is the main target in typical atrial flutter ablation [10, 22]. Studies have previously shown that RMN is safe and effective for ablation of typical atrial flutter compared to manual ablation [12, 23, 24]. While macro-reentrant atypical atrial flutter is generally related to atrial scar, fibrosis, surgery, and ablation, it is often resistant to antiarrhythmic drugs and might be cured by manual ablation [2, 22]. However, the effectiveness of RMN on atypical atrial flutter was uncertain.

In the current study, we found that RMN could be applied for PMAFL ablation, either for MI line or AS linear ablation. The acute and long-term success rate of MI linear ablation using RMN for PMAFL was lower when compared to manual ablation, which may be due to epicardial or CS ablation not being performed [8, 22]. However, AS linear ablation with RMN showed similar outcomes to manual ablation for PMAFL [8, 19, 25]. There are several advantages of the anterior septal line for PMAFL using RMN. First, as the soft magnetic catheter guided by RMN is quite stable and easy to control with micromovements in the anterior septal area, a bidirectional conduction block is routinely feasible to achieve. In contrast, it is often difficult to obtain adequate contact without a steerable sheath by the manual catheter ablation technique for the AS line [15]. Second, RMN-guided ablation in the AS line area is safe and effective. Finally, the fluoroscopy time is shorter with AS linear ablation guided by RMN.

### 4.5. Limitations

First, this is a single-center study reporting ablation of PMAFLs using RMN, and case numbers are relatively low. Accordingly, further large-scale, multicenter studies are required to validate the results. Second, epicardial ablation or ablation in CS was not performed in the MI group to avoid complications, which might decrease the acute success rate of the MI group. Third, as a majority of the PMAFL patients presented with AF, PVI was performed either previously or simultaneously for these patients. As a result, the procedure data do not represent results for PMAFL ablation only, and the long-term outcome might have bias. Finally, this study is limited by the inherent nature of a retrospective study, and prospective studies are needed.

## 5. Conclusions

We determined it was feasible to achieve a bidirectional conduction block on the anterior septum with the use of RMN. Additionally, AS linear ablation could be a more effective approach for treating PMAFL than MI linear ablation. Therefore, our data suggest that RMN-guided ablation in the AS line area is safe and effective for the treatment of PMAFL.

## Figures and Tables

**Table 1:** Baseline characteristics of patients.

Parameters	MI line (*n* = 10)	AS line (*n* = 26)	Total (*n* = 36)	*P* value
Age (years)	63.8 ± 16.4	63.1 ± 7.5	63.3 ± 10.5	0.863
Gender (female, %)	2 (20.0%)	6 (23.1%)	8 (22.2%)	0.842
Smoking	5 (50.0%)	9 (34.6%)	14 (38.9%)	0.396
Alcohol	5 (50.0%)	11 (42.3%)	16 (44.4%)	0.677
Ejection fraction	62.5 ± 4.8	61.1 ± 8.4	61.5 ± 7.5	0.638
PM	2 (20.0%)	3 (11.5%)	5 (13.9%)	0.511
AFL	6 (60.0%)	6 (23.1%)	12 (33.3%)	0.035
AFL + AF	4 (40.0%)	20 (76.9%)	24 (66.7%)	0.035
PVI history	4 (40.0%)	19 (73.1%)	23 (63.9%)	0.064
PVI times	1 ± 1.3	0.9 ± 0.7	0.9 ± 0.9	0.865
Diseases				
HBP (*N*, %)	7 (70.0%)	14 (53.8%)	21 (58.3%)	0.379
SHD (*N*, %)	3 (30.0%)	4 (15.4%)	7 (19.4%)	0.321
T_2_DM (*N*, %)	1 (10.0%)	4 (15.4%)	5 (13.9%)	0.676

AF, atrial fibrillation; AFL, atrial flutter; AS, anterior septal; DM, diabetes; HBP, hypertension; MI, mitral isthmus; PM, pacemaker; PVI, pulmonary vein antrum isolation; SHD, structural heart disease.

**Table 2:** Procedural outcome.

Parameters	MI line (*n* = 10)	AS line (*n* = 26)	Total (*n* = 36)	*P* value
Procedure time (min)	148.7 ± 46.1	123.2 ± 30.1	130.2 ± 36.4	0.058
RFCA time (min)	25.9 ± 11.4	23.5 ± 12.6	24.2 ± 12.2	0.609
Fluoroscopy time (min)	8.0 ± 4.4	5.1 ± 2.7	5.9 ± 3.5	0.024
X-ray dose (Gycm^2^)	9.9 ± 7.9	4.8 ± 3.1	6.2 ± 5.3	0.077
Complication	1 (10.0%)	1 (3.8%)	2 (5.6%)	0.470
Heparin (U)	7050.0 ± 1571.4	6807.7 ± 1428.8	6875.0 ± 1451.0	0.660
Fentanyl (U)	317.2 ± 153.1	244.3 ± 100.9	265.5 ± 120.4	0.128

AS, anterior septal; MI, mitral isthmus.

**Table 3:** Acute results and long-term follow-up.

Parameters	MI line (*n* = 10)	AS line (*n* = 26)	Total (*n* = 36)	*P* value
Acute success rate	7 (70.0%)	25 (96.2%)	32 (88.9%)	0.025
Follow-up (month)	42.3 ± 19.3	35.5 ± 21.0	37.4 ± 20.5	0.383
Total recurrences	3 (30%)	7 (26.9%)	10 (27.8%)	0.854
AFL success rate	3/6 (50.0%)	6/6 (100.0%)	8/12 (75.0%)	0.046
Long-term success rate	4 (40.0%)	19 (73.1%)	23 (63.9%)	0.049

AS, anterior septal; MI, mitral isthmus.

## Data Availability

Data are available on request.
